# The Effect of Biorelevant Hydrodynamic Conditions on Drug Dissolution from Extended-Release Tablets in the Dynamic Colon Model

**DOI:** 10.3390/pharmaceutics14102193

**Published:** 2022-10-14

**Authors:** Connor O’Farrell, Mark J. H. Simmons, Hannah K. Batchelor, Konstantinos Stamatopoulos

**Affiliations:** 1 School of Chemical Engineering, University of Birmingham, Edgbaston, Birmingham B15 2TT, UK; 2 Strathclyde Institute of Pharmacy and Biomedical Sciences, University of Strathclyde, 161 Cathedral Street, Glasgow G4 0RE, UK; 3 Biopharmaceutics, DPD, MDS, GSK, David Jack Centre, Park Road, Ware, Hertfordshire SG12 0DP, UK

**Keywords:** dynamic colon model (DCM), large intestine, colon, peristalsis, colonic motility, in vitro model, dissolution, USPII, extended release, colon targeted drug delivery

## Abstract

The in vitro release of theophylline from an extended-release dosage form was studied under different hydrodynamic conditions in a United States Pharmacopoeial (USP) dissolution system II and a bespoke in vitro tubular model of the human colon, the Dynamic Colon Model (DCM). Five biorelevant motility patterns extracted from in vivo data were applied to the DCM, mimicking the human proximal colon under baseline conditions and following stimulation using polyethylene glycol or maltose; these represent the lower and upper bounds of motility normally expected in vivo. In the USPII, tablet dissolution was affected by changing hydrodynamic conditions at different agitation speeds of 25, 50 and 100 rpm. Applying different motility patterns in the DCM affected the dissolution profiles produced, with theophylline release at 24 h ranging from 56.74 ± 2.00% (baseline) to 96.74 ± 9.63% (maltose-stimulated). The concentration profiles of theophylline were markedly localized when measured at different segments of the DCM tube, highlighting the importance of a segmented lumen in intestine models and in generating spatial information to support simple temporal dissolution profiles. The results suggested that the shear stresses invoked by the unstimulated, healthy adult human colon may be lower than those in the USPII at 25 rpm and thus insufficient to achieve total release of a therapeutic compound from a hydroxyethyl cellulose matrix. When operated under stimulated conditions, drug release in the DCM was between that achieved at 25 and 50 rpm in the USPII.

## 1. Introduction

Understanding and replicating the gastro-intestinal environment using in vitro and in silico models is of great importance for the development and evaluation of orally administered dosage forms. Whilst the hydrodynamic conditions within the GI tract are an important factor governing drug release, they are not replicated in commonly used dissolution apparatus, such as the ubiquitous United States Pharmacopoeial (USP) dissolution system II. This comprises a cylindrical vessel with a hemispherical base and a vortex-inducing paddle. Although some apparatus have been developed to change pH dynamically in accordance with transit through the GI tract [1,2], there has again been no attempt to replicate the hydrodynamic conditions [3].

Some dosage forms are designed to release drug over an extended period of time, or to reach specific regions of the GI tract. To achieve this, it is common to include polymeric excipients that serve to control release by erosion, which is driven by hydrodynamics [4]. Historically, the rate of drug release from polymeric matrices has been calculated using the power law model by Korsmeyer and Peppas (1983) (Equation (1)).

(1) $$\frac{M_{t}}{M_{\propto }}=Kt^{m}\;\;\;\;\;$$

Here, the ratio of $M_{t}$ to $M_{\propto }$ denotes the remaining fraction of the initial mass of undissolved drug at time t and K is the rate constant that depends upon the micro- and macro-structural features of the dosage form. The exponent, m, is used to characterise the different mechanisms of release.

**Figure 1:**
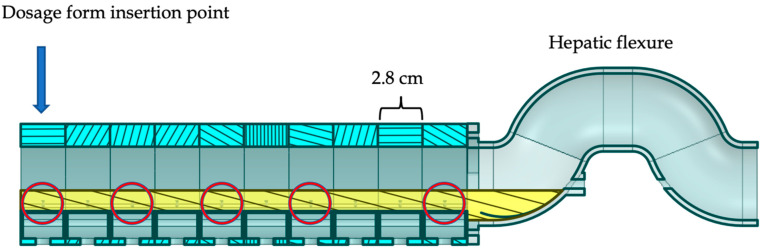
Cross section of the DCM partially filled with 100 mL fluid. Circled are the sample ports (SP) SP1, SP3, SP5, SP7 and SP10 from left to right. The DCM is comprised of 10 identical, modular segments of length 2.8 cm. A dosage form is introduced at segment (S) S1. Adjacent to the final segment, S10 at the opposite end of the tube is adjacent to the mimic hepatic flexure.

The dynamic colon model (DCM), shown in Figure 1, is an advanced in vitro model of the human proximal colon that replicates segmental peristaltic motility [5]. It is the only model known to the authors to reproduce peristaltic motility of the colon in a segmented luminal architecture. The DCM can be programmed to mimic different intestinal motility patterns observed in vivo. This permits in vitro analysis of different motility parameters such as occlusion degree and rate, isolated single contractions versus wave contractions and direction of propagation. Previously, the dissolution of theophylline from an extended-release dosage form in the DCM was studied under application of a high amplitude pressure wave (HAPW) [6]. The HAPW was applied at a frequency of 5 min over a duration of 8 h. However, this aggressive motility represented a highly extreme version of the in vivo situation, where a HAPW occurs only 4–10 times per 24 h in the unprepared human colon [7,8,9].

In addition, a low amplitude cyclic antegrade propagating wave (CPPW) travelling the 28 cm length of the DCM (mimicking from caecum to hepatic flexure) has been used in previous studies [10,11,12]. Its motility was based on observations from a high resolution manometry study on healthy volunteers by Dinning et al. [13]. CPPWs have also been reported to propagate in the retrograde direction [13].

In recent years, both high-resolution manometry and magnetic resonance imaging (MRI) have illustrated the variability of peristaltic contractions inside the colon. These include three patterns observed using cine-magnetic resonance imaging (cine-MRI) of healthy adult human subjects at a resting ‘baseline’ state and under stimulation by oral administration of either polyethylene glycol (PEG) electrolyte solution or maltose [12,14,15]. These motility patterns have also been applied to an in silico model of the human proximal colon as reported by Schutt et al. [16]. Simulated colonic motor patterns with the highest frequency of single shear stress peaks were shown to cause a higher rate of drug release from a solid oral dosage form, whilst dampened motility led to a more pronounced influence of diffusional drug release [16]. However, these simulations were based upon a homogenous dosage form modelled using a lattice spring model of solid particles, rather than the complexity of a real tablet with a range of excipients and particle-particle or particle-fluid interactions within the overall structure. This limitation is of particular relevance when considering an erodible modified release tablet that would be applied to the colon.

By simulating physiologically derived motor patterns in vitro, it becomes possible to understand the capability of a patient’s colonic motor patterns to influence release of a therapeutic from a drug delivery vehicle. Furthermore, it facilitates assessment of the sensitivity or resistance of the formulation to the shear forces that are likely to be present in the human colon. The aim of this study is to evaluate the effect of simulated colonic motor patterns within the DCM on the dissolution of theophylline from an erodible extended-release formulation. This has required the integration of the new motility profiles into the DCM and the application of these motility profiles to the tablet of interest (Uniphyllin Continus^®^).

## 2. Materials and Methods

### 2.1. Materials

Sodium carboxymethyl cellulose (NaCMC) 700,000 MW and potassium phosphate mono- (KH_2_PO_4_) and dibasic (K_2_HPO_4_) were purchased from Merck. Uniphyllin Continus^®^ (UC) 200 mg prolonged release theophylline tablets (Napp Pharmaceuticals) were purchased from New Castle Healthcare NHS pharmacy, described in Table 1 [17,18].

### 2.2. Calibrating the DCM for Replication of Colonic Motor Patterns

To develop the motility patterns in the DCM using previously published in vivo data, it was necessary to first convert the available in vivo parameters, namely the occlusion degree and occlusion velocity, into a form that is useful for the DCM.

#### 2.2.1. Integrating Occlusion Degree

The DCM consists of ten segments, each of which comprises three haustra which combine to form a circular unit. When a segment contracts or relaxes, all three constituent haustra move synchronously. As in the human colon, the haustra of the DCM are convex in shape, so the occlusion degree varies along the length of a segment. The occlusion degree was calculated from Equation (2), using the ratio of cross-sectional area measured at the central apex of the haustra (i.e., the maximum occlusion degree reached in the segment), to the cross-sectional area at the neutral position.

(2) $$OD_{x}=100\left(1-\frac{A_{x}}{A_{N}}\right)\;\;\;\;\;$$

Within the DCM, the haustra are contracted or relaxed using a hydraulic system controlled by syringe drivers. An in-house software was used to set syringe displacement to 0, 5, 10, 20, 25 and 28 mm using a stepper motor at a constant syringe speed of 15 mm s^−1^, pausing for 5 s when a segment reached full contraction. An endoscopic camera fixed in position inside the DCM was used to capture the motion at 50 frames per second. At each position, the occlusion degree was calculated using MATLAB to find the area of the lumen in pixels after binarizing the images. This was conducted in triplicate for each degree of syringe displacement. Using the top of the membrane at the centre of the haustra as a point of reference, the displacement of the membrane was also measured, similar to the method used by Stamatopoulos et al. [12]. A linear relationship (Equation (3)) was established between syringe displacement (SD) and membrane displacement (MD) with a correlation coefficient of 0.996.

(3) MD=0.3425·SD+0.7246

Occlusion degree was found to increase linearly with syringe displacement (SD) over the range of occlusion degrees that have been reported in vivo 18 ± 10 and 59 ± 18% under baseline and stimulated conditions, respectively (Equation (4), with a correlation coefficient of 0.997).

(4) OD=5.797·SD−44.03

#### 2.2.2. Integrating Occlusion Velocity

The occlusion velocity was related to the displacement of the syringe that hydraulically inflated and deflated the haustra. An iPhone camera with a frame rate of 60 s^−1^ was clamped in position, with a single DCM segment in direct view that was adhered to a ruler with graduations of 1 mm. This set up was clamped and held at the identical elevation and orientation to the DCM. The in-house software was used to set syringe speed to 5, 10, 20, 25, 30, 40, 45 and 50 mm/s, and displacement to a constant 15 mm. The corresponding membrane speed for each syringe speed was measured and recorded for each of the three haustra in this segment.

A linear relationship (Equation (5)) was obtained between occlusion velocity (OV) and syringe speed (SS) with a correlation coefficient of 0.997.

(5) OV=0.3172·SS+0.9221

### 2.3. Application of the DCM Motility Patterns

**Figure 2:**
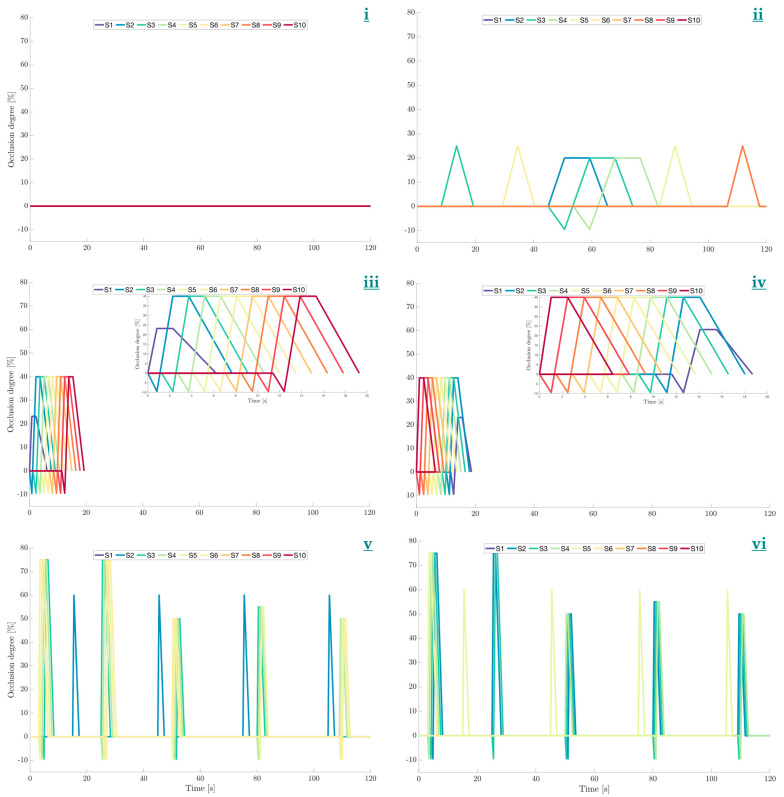
Motility patterns applied to the DCM. (**i**) Static, (**ii**) Baseline [12], (**iii**) Antegrade CPPW, (**iv**) Retrograde CPPW, (**v**) PEG stimulated motility and (**vi**) Maltose stimulated motility. Inset figures are placed in C and D to show the motility of each CPPW in more detail.

Table 2 shows the motility parameters for each pattern used, with the in vivo parameter shown for comparison. Figure 2 shows a graphical representation of the wall motion for each pattern. The patterns included a zero-motility state applied as a control. The motility patterns in this study were developed from analysis by Stamatopoulos et al. [12] of in vivo cine-MRI data produced by Marciani et al. and Hoad et al. [14,15] as well as using data from high resolution manometry [13]. All of these were also simulated computationally in [16], except for a retrograde version of the CPPW applied here.

Wave travel distance was measured as the distance between the start of the first segment contracting to the end of the last segment contracting. Thus., i.e., if one segment has a length of 2.8 cm (Figure 1), a wave that involves contraction of three adjacent segments travels a distance of 8.4 cm.

The time taken was calculated from the time that the first segment begins to contract to the time at which the final segment in the sequence reaches its point of maximum contraction. The repetition frequency of all patterns was set to 120 s as used by Schutt et al. [16], representative of the in vivo data presented in Stamatopoulos et al. [12]. Over the 24 h duration of the experiments, there were therefore 720 cycles per experiment.

The motility patterns contained propagating waves in either direction and isolated single contractions. However, they differed in the location of contraction with respect to the point of tablet introduction, occlusion degree and occlusion rate. Higher occlusion rates are associated with sharper peaks in recorded luminal pressure when the contents have a greater viscosity than water [6,19]. This causes more sudden disturbances to the fluid contents of the lumen, likely producing higher shear rates and therefore increased capacity for tablet erosion.

During a propagating contraction, the DCM replicated the sequential peristaltic wall motion that is well documented in vivo, wherein a segment remained contracted until the following segment reached full contraction. The wall then returned back to its neutral position at a 0.26-fold slower speed than the occluding event, to mimic the viscoelastic properties of the colon wall in line with in vivo observations [12]. This causes the pressure profile generated to tail off in line with in vivo high-resolution manometry [12,20]. This overall propagating wave motion of the wall has been shown to create a strong backflow inside the DCM [11]. Isolated single contractions were followed by immediate relaxation at the same ratio of 0.26 and 0.91 times the contraction speed in stimulated and baseline conditions, respectively. During a propagating contraction, the occlusion degree during relaxation was set constant at −10%, equating to a 3.5 mm outward displacement of the membrane and a syringe displacement of −6 mm.

Figure 2 shows the occluding behaviour of each motility pattern used in this study. Each segment is colour coded, with the warmer colours tending towards the caecum, S1 being red, moving through cooler colours culminating in purple for S10. Therefore, adjacent contractions that transition to warmer and cooler colours with increasing distance from the caecum represent antegrade and retrograde propagating waves, respectively.

### 2.4. Motility Index

The motility index (MI) is a parameter established by Marciani et al. [14] for the purpose of comparing the level of colonic motility observed between different subjects and stimulated versus unstimulated conditions using cine-MRI sequences. This was described using Equation (6), where $K=\frac{t}{t_{iv}}$. t represents the duration of the time window that is to be analysed. This window is broken down into smaller episodes of constant duration t_iv_. Based on the motility observed, Marciani et al. [14] chose to analyse 20 s intervals within a period of 120 s (K=6).

(6) $$MI=\overset{K}{\underset{k=1}{\sum }}{\left(t_{iv}\cdot N_{seg}\right)}_{k}$$

The number of segments, *N*_seg_, was also derived from analysis of MRI data and depended on visible motility and interpatient variability. Considering in vitro and in silico models, the number of segments is well-defined in the geometry of the system and does not rely on imaging capabilities and unpredictable in vivo motility to distinguish between segments. Stamatopoulos et al. [6] and Arkwright et al. [19] demonstrated occlusion velocity to be key in generating pressure during non-occluding events. Therefore, a modified version of the motility index, MI_DCM_ (described by Equation (7)), was introduced, whose derivation is given in Appendix A. The MI_DCM_ is a dimensionless index that is sensitive to both occlusion velocity and frequency of contractile activity that causes individual peaks in shear stress, which Schutt et al. [16] reported to be an effective predictor of dissolution ability. MI and MI_DCM_ values for each pattern are presented in Table 3.

(7) $$MI_{DCM}=\frac{1}{\omega \cdot L}\overset{K}{\underset{k=1}{\sum }}{\left(v\cdot N_{seg}\right)}_{k}$$

### 2.5. Dissolution Experiments

#### 2.5.1. Analysis of Drug Concentration

Drug concentration was measured by UV-Visible spectrophotometry (Biochrom Libra S12) at 270 nm using a quartz cuvette with 10 mm optical path at 22 °C. Absorbance at 270 nm was compared against a calibration curve that was linear between 2–20 μg mL^−1^ (correlation coefficient of 0.999) obtained through testing a serial dilution of standard solutions (N = 4).

#### 2.5.2. Dissolution Experiments in the USPII

Preliminary dissolution experiments (N = 4) were conducted in the Agilent 708-DS USPII system (Agilent Technologies, Santa Clara, CA, USA) using conventional 1 L capacity vessels to verify the shear sensitivity of the tablets. Agitation speed was fixed at either 25, 50 and 100 rpm. USPII vessels were filled with 900 mL pH 7.4 aqueous phosphate buffer. Tablets were simultaneously dropped into the vessels using a dosage drop device (Agilent Technologies, USA). The temperature of the vessels was maintained at 37 ± 0.1 °C using an Agilent 708-DS USPII water bath. One sample location was used per vessel as per USP chapter <711> Dissolution [21]. Samples 1 mL in size were removed at 0.25, 0.5, 1, 2, 3, 4, 6, 8, 10, 12, 16 and 24 h and filtered using 0.45 μm polyethersulfone (PES) syringe filter membranes (Whatman, Cytiva, Marlborough, MA, USA) before storage in amber vials for a maximum of 24 h until analysis. Dissolution media was replenished immediately after sample withdrawal using the 850-DS automated sampling system (Agilent Technologies, Santa Clara, CA, USA).

#### 2.5.3. Dissolution Experiments in the DCM

The DCM was partially filled with $V_{L}$ = 100 mL of 0.25% (*w/w*) NaCMC solution adjusted to pH 7.4 using phosphate buffer as illustrated in Figure 1, in line with previously published dissolution experiments [6]. Theophylline is highly soluble in water and does not exhibit a strongly pH-dependent dissolution within the pH range observed in the human proximal colon [17,18]. The temperature of the fluid inside the DCM lumen was maintained at 37 ± 0.3 °C. The tablet was directly inserted into the ileocecal port of the DCM, highlighted in Figure 1. Immediately following tablet insertion, a motility pattern (see details in Table 1) was applied and repeated at a frequency of one cycle per 120 s lasting 24 h. At 0.25, 0.5, 1, 2, 3, 4, 6, 8, 10, 12, 16 and 24 h, $V_{r}$= 1 mL of sample fluid was withdrawn from 5 different sample ports (SP) simultaneously along the length of the DCM, SP1, SP3, SP5, SP7 and SP10, circled in Figure 1. Samples were filtered using a 0.45 μm PES syringe filter and analysed.

Over the course of the experiment, 5 mL was removed and replenished at each of the eleven timepoints, corresponding to 55% turnover of dissolution media, this needed to be accounted for in the analysis of cumulative drug release and the methodology used as shown in Section 2.5.4.

#### 2.5.4. Dissolution Profile Calculations

The DCM was oriented horizontally, in the supine position typically assumed by subjects during the manometric, scintigraphic and MRI procedures that informed design of the model [6,11,12]. Thus, the fluid was distributed throughout the lumen such that 8.9 mL fluid was present in each of the ten segments and 11 mL fluid resided in the hepatic flexure (see Figure 1). The assumption was made that the concentration of dissolved theophylline was homogeneous within a segment. The volume of each segment after sampling $V_{S}$, was 8.5 mL with $V_{HF}=10.45\;\mathrm{mL}\;$in the hepatic flexure.

The result, denoted $C_{S\left(t\right)}$, was the measured concentration, C, in segment s at time t. Firstly, this value was used to estimate the mass of dissolved theophylline removed from the system by the sampling procedure at segment S and time t, $m_{S\left(t\right)}$ using Equation (8).

(8) $$m_{S\left(t\right)}=V_{r}C_{S\left(t\right)}$$

This equation makes the reasonable assumption that the concentration of dissolved theophylline was homogeneous within a 1 mL sample vial. Therefore, the total mass of dissolved drug removed from the DCM lumen $m_{sampled}$ at timepoint t was given by Equation (9):

(9) $$m_{sampled,\left(t\right)}=\overset{S\;=\;10}{\underset{S\;=\;1}{\sum }}m_{S\left(t\right)}$$

where S∈1,3,5,7,10 (i.e., each of the five sample ports used). Over the course of the experiment, the cumulative sum of mass removed was used to estimate the total dissolved drug that had been removed in saturated sample fluid until that time point. Therefore, the cumulative sum of mass removed, $m_{sampled,\left(T\right)}$ from t = 0 until t = T resulted in the total mass removed, $m_{removed\;\left(T\right)}$, from the lumen up until time point T (Equation (10)).

(10) $$m_{removed\;\left(T\right)}\;=\;m_{sampled,\left(T\right)}+\overset{t\;=\;T-1}{\underset{t\;=\;0}{\sum }}m_{sampled,\left(T\right)}$$

The mass of dissolved drug residing in each sampled segment was given by Equation (11):

(11) $$m_{S\left(t\right)}=V_{S}C_{S\left(t\right)}$$

By plotting the concentration profile along the length of the DCM at each timepoint, the distribution of dissolved drug was modelled and the concentration in intermediate segments was estimated. The total drug present in the lumen at time t, $m_{L\left(t\right)}$, was given by Equation (12):

(12) $$m_{L\left(t\right)}=\overset{S\;=\;10}{\underset{S=1}{\sum }}m_{S\left(t\right)}+C_{HF\left(t\right)}V_{HF}$$

where $C_{HF\left(t\right)}$ is the concentration of dissolved drug estimated in the hepatic flexure at time t. Ultimately, to calculate the fractional drug release and fit to a power law model, the remaining mass of undissolved drug $M_{t}$ was given by Equation (13):

(13) $$M_{t}=m_{L\left(t\right)}+\;m_{removed\;\left(t\right)}$$

#### 2.5.5. Comparison of Dissolution Profiles

The model-independent dissimilarity factor, *f*_1_, described by Equation (14), was applied to analyse the difference between two dissolution profiles at each time point [22]. Two curves are deemed similar if 0 ≤ *f*_1_ ≤ 15 (%).

(14) $$f_{1}\;=\;100\times \left\{\frac{\left[\sum_{t\;=\;1}^{n}\left|R_{t}-T_{t}\right|\right]}{\sum_{t\;=\;1}^{n}R_{t}}\right\}$$

The similarity between dissolution curves was tested by the model-independent similarity factor, $f_{2}$, approach (Equation (15)), where $50\leq f_{2}\leq 100$ suggests that two dissolution profiles are similar [23]. An *f*_2_ value of 50 means that there is an average of 10% difference between mean dissolution (%) at all specified time points [24].

(15) $$f_{2}\;=\;50\log\left[\frac{100}{1+\sqrt{\frac{\sum_{t\;=\;1}^{t\;=\;n}{\left(A_{t,X}-A_{t,Y}\right)}^{2}}{n}}}\right]\;$$

where $A_{t,X}$ and $A_{t,Y}$ are the% dissolution of theophylline at time t under motility pattern X and Y, respectively in the DCM, or between rpm values X and Y in the USPII. n denotes the number of time points compared in the profiles. Only datapoints 15 < *X* < 85% release were used to maintain validity of this method [24].

Additionally, one-way analysis of variance (ANOVA) was used to test for significant differences (*p* < 0.05) between release (%) of theophylline at individual time points.

#### 2.5.6. Tablet Location

Using an endoscope camera, the segmental position of the tablet in the DCM was recorded after the dissolution experiment was completed.

## 3. Results and Discussion

### 3.1. Motility

When establishing the parameters for motility, the primary concern was ensuring that the parameters chosen were within the observed ranges in vivo. However, technical limitations of the DCM meant that not all in vivo parameters could be matched. For example, when stimulated (using PEG or maltose), occlusion velocity was reported to be 36 ± 1.7 mm s^−1^ in vivo. However, this correlated to a mean syringe speed of 109.63 mm s^−1^ which is beyond the capabilities of the stepper motors employed. The maximum operable syringe speed for the syringe distances travelled in this study was 38 mm s^−1^. However, previous experiments have demonstrated that pressures within the range of those measured using high resolution catheters in the ascending colon in vivo have been recorded for non-occluding events in the DCM at occlusion rates as low as 4.3 mm s^−1^ [13,25]. Additionally, occlusion velocities this high may only occur a few times per day in vivo. Therefore, the pressure events generated by the motility patterns in this study were considered to be biorelevant.

### 3.2. USPII Dissolution Profiles

**Figure 3:**
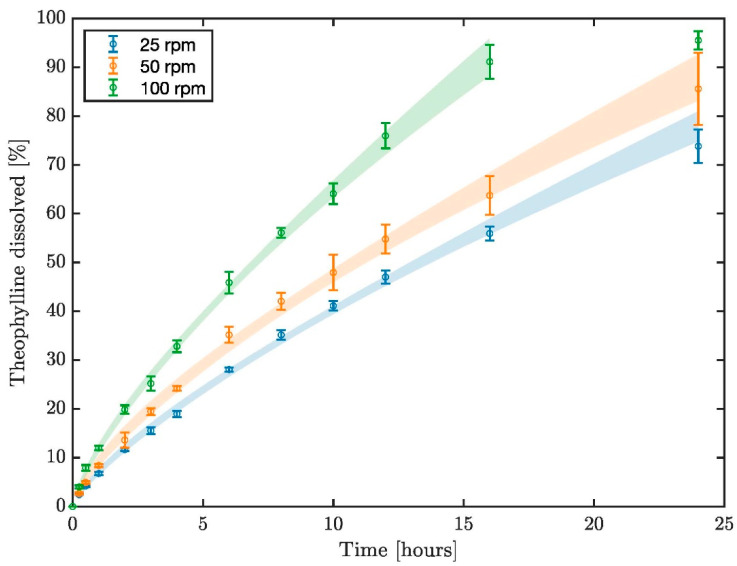
Dissolution profiles of theophylline from Uniphyllin Continus (UC) 200 mg tablets in the USPII apparatus at 25, 50 and 100 rpm.

Figure 3 presents dissolution profiles of theophylline from the UC tablet in the USPII at different agitation speeds. The profile generated at 25 rpm is significantly different to those obtained at 50 and 100 rpm: *f*_1_ = 18.2 and 55.7%, *f*_2_ = 56.1 and 33.0%, respectively. The profiles generated at 50 and 100 rpm were also significantly different with *f*_1_ = 31.8 and *f*_2_ = 45.0%. Dissolution rate and drug release increase with rpm, demonstrating that the overall release and the release rate are sensitive to shear rate. The consequent erosion of the gel layer contributes significantly to release from these HEC matrix formulations.

### 3.3. Tablet Position

When the static and baseline motility patterns were applied, the tablet remained in the same position throughout, therefore the base of the tablet was not exposed to fluid. Conversely, when the CPPWs and stimulated patterns were used, the orientation of the tablet had changed after the experiment. Insertion of the intact tablet at the ileocaecal junction assumes that the dosage form reaches the proximal colon intact which is an unlikely situation in vivo. Nevertheless, the impact of the different in vivo-relevant motility patterns on dissolution behaviour of an erodible tablet has been demonstrated.

### 3.4. Segmental DCM Dissolution Profiles

**Figure 4:**
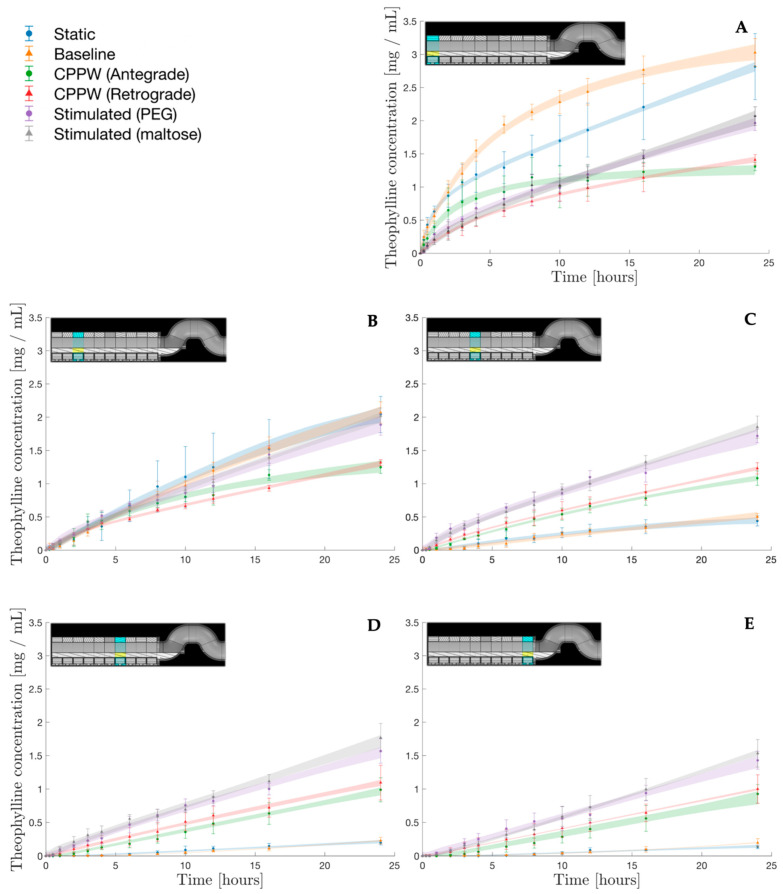
Concentration profiles of theophylline in segments S1 (**A**), S3 (**B**), S5 (**C**), S7 (**D**) and S10 (**E**) of the DCM under different motility patterns.

Standard drug dissolution/release profiles in the pharmaceutical industry are presented as a single profile with no spatial information, as in Figure 3. However, the lumen of the DCM, much like the intestine in vivo, is not perfectly mixed. This means that information to describe how drug concentration varies with location is key to understanding how much API has been released from the tablet and how effective the motility pattern is at distributing the dissolved compound. Samples were withdrawn from five locations along the DCM to obtain the distribution of dissolved API along the DCM tube over 24 h. Figure 4 presents the concentration profiles of dissolved theophylline measured at S1, S3, S5, S7 and S10, progressively further from the point of tablet insertion, whilst the respective inset figure shows the location of the sampled segment.

Besides from the DCM, the architecture of most advanced in vitro models of the colon takes the form of a single tube [5]. The most advanced physiologically based pharmacokinetic (PBPK) models use oversimplified first order transit rate models that consider the colon to be a single homogeneously mixed compartment. It is well-documented that the colon contracts in a segmental fashion wherein there is likely to be regional differences in both mixing behaviour and thus drug concentration. This has been demonstrated using the DCM in this study; where mimicked in vivo motility involved frequent contractions (to different degrees of occlusion) whilst other segments remained stationary. The spatiotemporal information in Figure 4 demonstrates clearly that a drug concentration profile is highly dependent on the segments of the DCM lumen from which measurements are taken (Figure 4). If the DCM were a perfectly mixed system each segment should yield 2 mg mL^−1^ of theophylline. This demonstrates the importance of a segmented lumen in models of the colon, thus, there is a need to update the PBPK models to include a segmented colon. Furthermore, the segmental and cumulative dissolution data from the DCM could be integrated into PBPK models of the colon to better predict overall exposure; particularly for erodible formulations where motility is critical for drug release. This is relevant as it was observed that operating motility patterns with a low MI_DCM_ can cause the contents of some segments to be somewhat stagnant. Operating patterns with such low motility may therefore encounter potential solubility effects on the rate of release measured in segments containing the tablet body, especially considering vehicles with a poorly soluble payload.

S1 was the point of tablet insertion. Figure 4A presents the concentration profiles of theophylline measured at S1, representing measured dissolution in the fluid immediately surrounding the tablet, which did not move from its insertion point for the duration of the experiments. The highest concentration reached in S1 was under static (2.82 ± 0.50 mg mL^−1^) and baseline (3.07 ± 0.21 mg mL^−1^) conditions. When the DCM was static, drug release from the dosage form and distribution along the DCM was driven primarily by diffusion. This led to accumulation around the point of tablet insertion and high local drug concentration. Mass transport in the static model was not purely diffusive however, since the media sampling and replenishment process introduced advective mixing, although this was minimised by operating sampling/replenishment syringe pumps at low flow rates (6 mL min^−1^). Under baseline conditions, there were single, isolated contractions in S3, S6, S5 and S8, in chronological order. Additionally, there was a propagating pressure wave (PPW) from S2 to S4. This baseline contractile activity had a low MI_DCM_, involving low occlusion degrees (25%) and slow occlusion rates (1.5 mm s^−1^) compared to the other patterns (refer to Table 2). Under such lethargic wall motion, it is unlikely that sufficient energy was imparted to the fluid to mix the area of high drug accumulation in S1, with the segments of the DCM closer to the hepatic flexure via advective transport through the viscous dissolution media. This was proven by the very low concentrations reached in S5, S7 and S10 (Figure 4C–E), where the baseline and static concentration profiles were very similar and significantly lower than in all other motility conditions.

Furthermore, it is possible that the baseline contractions promoted back-mixing rather than antegrade propagation of the contents. However, it is evident that the rate of increase of concentration in S1 was higher under baseline conditions compared to static. This suggests that the local mixing caused by proximate wall motion; the PPW from S2 to S4 and the isolated contraction at S3, was sufficient to either impart sufficient shear stress to erode the outer layers of the dosage form or mix the contents within S1. To elaborate on the latter, it was assumed that the concentration within a segment was homogeneous. However, the robustness of this assumption is proportional to the mixing of the system; in a perfectly mixed system it holds true whilst in a stagnant system, such as the static DCM, it is less valid. The baseline pattern may have been sufficient to disturb the fluid surrounding the tablet and mix the contents of S1 such that the sample withdrawn from SP1 gave a more accurate reflection of how much drug had dissolved from the tablet. It is likely that there was a steep concentration gradient radiating from the tablet body outwards in these patterns with low MI_DCM_, exacerbated by the viscosity of the dissolution media used. This could mean that the highest concentrations in S1 under the low MI_DCM_ static and baseline conditions were not reflected by the concentration measurements taken from SP1.

Although the static and baseline patterns showed the highest concentrations of dissolved theophylline in S1 (Figure 4), these data also exhibited the highest variability. This could be due to a limitation of the UV-VIS methodology used where samples with a higher concentration were diluted to a higher extent, which increases the scale of any initial error due to an increased number of measurements required using the pipette.

Theophylline concentrations in S1 were generally lowest under the CPPWs, reaching 1.31 ± 0.06 and 1.41 ± 0.08 mg mL^−1^ at 24 h under the antegrade and retrograde waves, respectively. Figure 4 clearly shows a constant increase in concentration at S3, S5, S7 and S10 for the antegrade and retrograde CPPWs. After only 1 h, concentration of theophylline measured at S5, S7 and S10 was significantly higher than for the baseline pattern for both CPPWs. This clearly demonstrates that these patterns were effective at mixing along the length of the tube, significantly more so than the baseline motility pattern. Concentrations at S1 after 24 h were not significantly different between the antegrade and retrograde CPPWs. Interestingly however, there were significant differences in the course taken by the concentration profiles. The rate of increase in theophylline concentration measured at S1 was much higher for the antegrade pattern until t = 10 h, where a plateau was reached whilst the concentration profile generated by the retrograde CPPW followed the same trajectory (at a lower rate of increase) as the stimulated patterns until t = 8 h. This suggests that the antegrade wave may not have been as effective as the retrograde wave in driving advective transport of dissolved drug along the DCM tube, away from the tablet lying in S1. This is reinforced by the consistently lower mean drug concentrations measured at S5, S7 and S10, however the differences were not statistically significant (*p* < 0.05). Additionally, this hypothesis is supported by a previous MRI study of the DCM that showed that the highest magnitude velocities generated by an antegrade CPPW were backflows (retrograde) rather than in the direction of wave propagation [11].

The rate of increase of drug concentration at S1 followed similar trajectory to the retrograde CPPW, lower than the static, baseline and antegrade patterns, until t = 8 h. At this point, the concentration continued to increase at an almost constant rate until the peak at 24 h. The concentration at S3, S5, S7 and S10 also constantly increases over the duration of the experiment, consistently reaching higher values than in the CPPWs. This infers that in addition to extensive mixing capabilities, the hydrodynamics caused by the higher MI_DCM_ stimulant-driven patterns generated a higher erosive effect than the CPPWs, constantly releasing and dissolving drug from the tablet body and mixing it throughout the DCM.

At S1 after 24 h, the concentration of theophylline measured under application of the stimulated patterns lied in between the static and baseline patterns and the CPPWs, at 1.96 ± 0.11 and 2.07 ± 0.14 for PEG and maltose, respectively. These values are close to 2 mg mL^−1^ which would be expected for a homogenously mixed lumen and complete drug release. At S10 however, drug concentration was lower, reaching 1.43 ± 0.13 and 1.53 ± 0.21, therefore this situation was not realised. The highest release measured at S7 and S10 was from the Maltose stimulated pattern, closely followed by the PEG stimulated pattern. This demonstrates that the stimulated motility patterns were effective at distributing dissolved drug throughout the DCM. In these patterns with the highest MI_DCM_, wave propagation speed and frequency were higher, though wave propagation distance was shorter, travelling a maximum of 4 segments compared to 10 in the CPPWs. This proves that inside the DCM it is not necessary for a contractile wave to propagate the length of the system (such as in the CPPWs) to effectively transport and mix fluid contents across the entire length of the tube.

The concentration profiles generated by the stimulated motility patterns at S7 and S10 increased with time in a linear modality, as did that of the baseline and CPPW patterns. However, in the maltose stimulated motility pattern there was a discrepancy in the linear trend at t = 6 h. In the DCM, this type of artefact may be caused by dispersed tablet material that have become detached from the principal tablet body and were local to the sample point when samples were taken. The likelihood of this occurring is bound to increase with the eroding capabilities of the motility pattern. This type of discrepancy is likely to be more prevalent in the DCM than in typical USPII apparatus, due to a higher sample point to media volume ratio.

### 3.5. Cumulative Temporal Theophylline Release Profiles in the DCM

**Figure 5:**
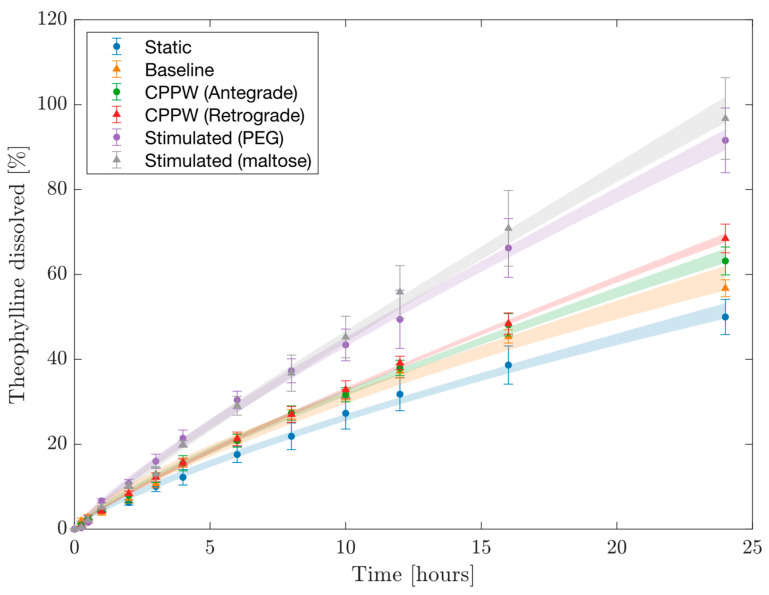
Cumulative release profiles in the DCM under different motility patterns. The shaded regions represent the 95% confidence interval around the mean value predicted by the model (Table 4).

The cumulative dissolution profile generated under each motility pattern is shown in Figure 5. This profile was built from the segmental concentration measurements, from which the distribution of the dissolved drug throughout the model could be estimated to evaluate the total mass of dissolved theophylline in the DCM lumen. The shaded regions represent the 95% confidence intervals for the power law models fitted to the experimental data, further explained in Table 4.

In the early stages of dissolution where t < 3 h, all profiles were similar to the static and baseline patterns, suggesting that diffusive mass transport governed release from the tablet. Subsequently, the importance of erosion becomes evident as the dissolution profiles under mimic stimulated conditions deviated significantly from the static and baseline waves. This is likely to align with hydration time of the HEC gel layer. From this point onwards, the hydrodynamics becomes important and the potential of the DCM to discriminate drug release profiles based upon physiologically relevant motility patterns is highly pertinent. The ability to recreate motility conditions demonstrates the power of the DCM to replicate inter- and intrasubject variability in motility, including extremes, and how this may influence colonic drug delivery. However, the authors recognise that in vivo the tablet would be subjected to the conditions of the upper GI tract prior to reaching the colon and that these aspects are not recreated in this study. A future study that included a biorelevant mimic of the conditions including the hydrodynamics of the upper GI tract that enabled delivery of the hydrated tablet without disruption of the swollen gel layer would be a great extension of this work.

The baseline motility was able to release 56.74 ± 2.00% of theophylline form the formulation over 24 h, significantly higher than achieved by the static DCM 49.98 ± 4.14%. However, the spatial information provided by Figure 4 showed that it was highly ineffective at transporting dissolved API along the DCM tube to segments beyond S3. When directly compared, the dissolution profiles generated from the antegrade and retrograde CPPWs were not statistically different (*f*_1_ = 5.5%, *f*_2_ = 76.4%). However, when compared to the profile measured under static conditions, the antegrade CPPW was not significantly different to the static profile (*f*_1_ = 19.4%, *f*_2_ = 54.3%) whilst the retrograde CPPW was different (*f*_1_ = 31.2%, *f*_2_ = 48.0%). This shows that the advective motion generated by a retrograde CPPW are sufficient to elevate the release and distribution of the drug inside the DCM beyond simple diffusion. However, the mean release achieved by the retrograde wave was significantly higher than the antegrade wave at t = 24 h. Although the wave parameters of the antegrade and retrograde contractions were equal, the propagation distance of the wave and the location of the wave front relative to the tablet position are drastically different. This is likely to be the leading contributing factor to the significantly higher mean release at t = 24 h under the retrograde CPPW. Additionally, O’Farrell et al. [5] showed the nature of a CPPW with 40% occlusion degree to cause high velocities in the opposite direction to wave propagation, which may be attributed to the increased rate of dissolved theophylline transport along the length of the DCM. Furthermore, in one experiment using the retrograde motility pattern, a large fragment of tablet had been eroded from the bulk tablet body and was located at the hepatic flexure from t = 16–24 h. This suggests that motility patterns which include more frequent retrograde propagating pressure waves may be more effective at achieving higher release of a therapeutic from a dosage form located towards the early stages of the proximal colon. Figure 5 shows a decrease in rate of increase of theophylline concentration from t = 3 h in both the antegrade and retrograde CPPWs, compared to the stimulated profiles which maintain a steady rate of increase. This suggests that insufficient shear was generated in the CPPWs to maintain the release rate observed in the profiles generated by the stimulated patterns. The same conclusion can be drawn from the baseline pattern. After t = 16 h, the shear stresses generated by the baseline and CPPWs appeared to be insufficient to cause breakdown and release of the drug that lies in the core of the tablet body, since the stimulated motility patterns produced significantly higher release than all others. The maltose stimulated pattern performed best in achieving highest release of theophylline.

Comparing the PEG and maltose stimulated patterns, there was no significant difference between the release profiles obtained (*f*_1_ = 6.00%, *f*_2_ = 70.70%). However, the mean concentration was consistently higher for the maltose-stimulated pattern at most time points. Although the PEG-stimulated pattern had a higher motility index, different features of the pattern may have caused the lower release. For example, S2 was the closest segment to the tablet insertion point in both patterns. However, in the PEG-stimulated pattern, S2 only exhibited isolated contractions, compared to the maltose-stimulated pattern, where S2 was involved in propagating wave contractions. This might suggest that the flows of the contents of the lumen generated by propagating waves are more effective at eroding a dosage form in the local vicinity. Furthermore, it has been shown that the location of the contents with respect to contractile activity is vital in determining the velocities imparted to that fluid and therefore the shear rates experienced [11]. Propagating pressure waves in the PEG-stimulated motility pattern involved S6 rather than S2 in the maltose-stimulated pattern. This may have caused lower peaks in flow around the tablet located at S1, since contractions in S6 are further from the tablet and more fluid lies in between the contracting segment and the tablet to dampen the flow.

The findings from this study align with those from the in silico study by Schutt et al.-that the mimicked motor patterns based on stimulants were significantly more effective at releasing a water-soluble drug from an erodible vehicle. Stimulated motility patterns had a higher MI_DCM_ and thus exhibited faster occlusion rates and a higher frequency of waves and single contractions, in addition to higher occlusion degrees. This means that there would be a higher number of peaks in shear stress per 120 s cycle; Schutt et al. [16] predicted that drug release from a computationally simulated dosage form was significantly impacted by the number of shear stress peaks. This is likely to also be a contributing mechanism in vitro. Higher occlusion rates have been shown to generate higher pressures inside the DCM [12], which (through conservation of energy) would cause higher velocities of the contents and subject a dosage form to more intense agitation and higher shear rates, achieving higher release. Comparisons between the hydrodynamics experienced by the tablet in this study and the in silico study are limited though, since the solid dosage form modelled by Schutt et al. [16] was neutrally buoyant whereas the UC tablets sunk in the DCM fluid and had less surface exposed to high shear rates in the fluid. Overall, the findings from this study suggest the in silico model may have value for testing whether a particular motility pattern, combinations of patterns or changes in motility parameters have a significant effect on drug release. This could be conducted as a first step to prioritise in vitro trials.

As previously discussed, the significant difference in release resulting from the change in rpm of the USPII apparatus demonstrated by Figure 3 shows that this formulation is shear-sensitive. However, the dissolution profiles inside the DCM in Figure 5 show that extended-release formulations that reach the colon may need to have greater sensitivity to lower shear rates. If a significant portion of the API remains in the vehicle when a prolonged release formulation reaches the colon, the polymer gel layer may be too resistant to shear to release sufficient API for effective treatment. It may be beneficial for formulators to use in vitro tools that model different regions of the GI tract for different stages of release, for example an inner core that is more sensitive to shear after 5.3 h, when a formulation typically reaches the ascending colon (median colon arrival time in healthy adult humans, 95% confidence interval 4.51–5.48 h [26]). From these findings, it may be more applicable run the USPII apparatus with a pulsed agitation, stepping between 0–25 rpm with intermediate steps representing shear rates expected in vivo. This is similar to the stress test device developed by Garbacz et al. [25] based on physiological pressures a dosage form may experience during GI transit. However, precise information regarding in vivo shear rates is not currently available [17]. Future work with the DCM should use a combination of baseline and stimulated patterns in the same dissolution experiment in order to more accurately reflect the range of motility a healthy human subject may experience day-to-day after ingesting a solid oral dosage form. It may also be possible to evaluate the difference of DCM orientation on baseline dissolution, by rotating the DCM through 0–90° at predetermined intervals to mimic the influence of gravity and human physical activity. This would likely cause a change in positioning or orientation of the tablet, exposing the side of the tablet that constantly in contact with the lower DCM wall in this study.

The knowledge gained from overall release profiles can also be used to further understand the segmental concentration profiles. Figure 4 showed plateauing behaviour of the S1 baseline concentration profile from t = 6 h, suggesting a decrease in the rate of theophylline release. However, it is clear from Figure 5 and Table 4 that total release was not close to 100%. Therefore, the plateau could be due to the introduction of solubility effects as local concentration gradients between the gel layer of the tablet and the surrounding fluid in S1 were diminished and the fluid approached the aqueous solubility of theophylline (5.5 mg mL^−1^). This would retard the rate of mass transport by diffusion. Another possibility is that diffusion from the outer layers of the tablet was complete and the rate of diffusion from the inner core was slow and crystalline theophylline was essentially ‘locked up’ and inaccessible without erosion of the outer layers, due to a low MI_DCM_.

A power law model was fitted to the overall release profiles obtained in the DCM under different motility patterns (Figure 5), with R^2^ > 0.999 for all profiles, as shown in Table 4. Similar to in the Korsmeyer-Peppas model, the exponent, m, generally increased with MI_DCM_ and is likely to be related to the frequency and intensity of shear experienced in an environment that exhibits colon-like pulsatile, peristaltic hydrodynamics. There is some discrepancy in this trend as the PEG stimulated pattern had a higher MI_DCM_ than the maltose stimulated pattern but a lower m and overall release. This suggests a contributing factor of the locality of the contractile activity with respect to the tablet, as previously discussed.

Table 5 presents the release comparison at t = 4, 10 and 24 h for each DCM motility pattern versus the USPII at 25, 50 and 100 rpm. Release at 4 h was generally higher in the USPII and discrepancy between the USPII and DCM increased with rpm, with 38.98–62.79% greater release under 100 rpm compared to the DCM. Overall discrepancy between the DCM and USPII was most extreme at t = 10 h, with the DCM showing >30% less release than the USPII when all motility patterns were compared at agitation speeds of 50 and 100 rpm. This indicates that the release rate in the USPII under these conditions was considerably higher between t = 4 and t = 10 h. After 24 h, the DCM had had sufficient time to reduce the gap between release achieved in the systems. The static, baseline and CPPWs never achieved higher release than the USPII at any agitation speed. At t = 4, 10 and 24 h, release was higher under mimic stimulated conditions in the DCM compared with the USPII operated at 25 rpm, however it is unrealistic that the human proximal colon would display this level of stimulated activity for a continuous period of 24 h. These findings suggest that operation of the USPII at 25 rpm and above generates hydrodynamics conditions that may be too intensive to be representative of unstimulated human colon. However, the stimulated colon may generate hydrodynamic conditions that lie somewhere between 25 and 50 rpm. This data builds upon previous conclusions that the use of > 50 rpm in the USPII is not recommended for colon-targeted dosage forms [10].

Table 4 highlights that the baseline and CPPW patterns did not exceed 70% release over 24 h, this suggests that the HEC gel layer in UC tablets may be overly resistant to the shear stresses that may be present in the unstimulated colon. Higher release was achieved in the USPII at 25, 50 and 100 rpm. Although conclusions cannot be drawn on the shear rates likely to have occurred from this comparison, since a constant shear is applied in the USPII and shear in the DCM has a high spatiotemporal dependence [10]. Previous MRI studies of the DCM found velocities to vary from −2.16–0.78 cm s^−1^ and shear rate fluctuating between 0–8 s^−1^ when slower CPPWs were applied (0.4 and 0.8 cm s^−1^) at the same volume and viscosity as in this study.

The variability in the overall release profile in the DCM was high compared to the USPII, likely due to the increased number of steps included in data processing. However, there was similarly high variability in direct measurements, e.g., the concentrations presented in Figure 4. This may better reflect the level of variation in vivo due to position of the tablet in relation to contractions and potential to experience elevated shear from being squeezed between contracted haustra, amongst many other factors.

Regarding use of the *f*_2_ approach to classify profiles as statistically indifferent/different from one another in this study, caution must be taken around the level of significance used. This method is typically used to compare dissolution profiles between products, rather than of the same product using different dissolution methods [22]. However, since the same formulation and dissolution media are used in all experiments and the dosage form is intended for extended release, it would be highly unusual for the product to exhibit >10% difference at the earlier stages of dissolution. Additionally, the majority of official guidance from governing bodies worldwide regarding acceptance criteria for *f*_2_ comparisons are for immediate release formulations rather than extended-release formulations where time is integral for matrix hydration, gel formation and erosion to take place.

## 4. Conclusions

The in vitro dissolution of theophylline from Uniphyllin Continus 200 mg ER tablets was assessed in the USPII and the dynamic colon model (DCM). In the DCM, different motor patterns previously identified in vivo were replicated using the DCM: baseline conditions for a healthy adult human, antegrade and retrograde cyclic propagating pressure waves (CPPWs) and the stimulated (using PEG or maltose) colon. The intensity of hydrodynamics induced by motility patterns was measured using a motility index that incorporated luminal occlusion rate and the number and frequency of segmental contractions.

Release from the ER tablets was sensitive to agitation speed in the USPII dissolution apparatus. The integration of biorelevant motility into the DCM provided predictions on the extent as well as variability of drug release that may be anticipated from an ER formulation subjected to intestinal hydrodynamics, as a function of motor pattern and position.

Mimicked baseline contractions were highly ineffective at advective transport of dissolved drug along the DCM tube and maximum release was > 20% lower than in the USPII at 25 rpm. Operating patterns with such low motility may encounter potential solubility effects on the rate of release measured in segments containing the tablet body, especially considering vehicles with a poorly soluble payload. Motility patterns which include more frequent retrograde propagating pressure waves may be more effective at achieving higher release of a therapeutic from a dosage form located towards the early stages of the proximal colon (compared to antegrade propagating waves). When mimicking the stimulated colon, a higher theophylline release was achieved than in the USPII at 25 rpm, although it is unrealistic to maintain these conditions for 24 h in vivo. Operation of the USPII at a constant rate of 25 rpm and above generates hydrodynamics conditions that may be too intensive to be representative of unstimulated human colon.

## Figures and Tables

**Table 1:** Formulation information.

Formulation	Brand	Lot	Dosage [mg]	Excipients
ER theophylline	Uniphyllin Continus^®^	251186	200	Hydroxyethyl cellulose, povidone (K25), magnesium stearate, cetostearyl alcohol, macrogol 6000, talc

**Table 2:** Motility parameters of the static, baseline, CPPW (A = Antegrade, R = Retrograde) and stimulated (PEG or maltose) motility patterns applied to the DCM in this study, including the in vivo range where in vivo data is available.

Parameter + In Vivo Data if Available/Applicable	Static	Baseline	CPPW	Stimulated (PEG)	Stimulated (Maltose)
Single Contraction					
Occlusion degree (%)	0	25	-	60	60
Membrane displacement (mm)	0	4.80	-	6.87	6.87
Occlusion velocity (mm/s)	0	1.55	-	12.98	12.98
Syringe displacement (mm)	0	11.91	-	17.95	17.95
Syringe velocity (mm/s)	0	1.97	-	38	38
Wave					
Occlusion degree (%) ▪ Stimulated = 59 ± 18 ▪ Baseline = 18 ± 10	0	20	40.00 (S1: 23.15)	▪ A: 75, 55, 50 ▪ R: 75, 50	▪ A: 75, 55, 50 ▪ R: 75, 50
Membrane displacement (mm)	0	4.51	7.12 (S1: 23.99)	▪ A: 7.76, 6.58, 6.28 ▪ R: 7.76, 6.28	▪ A: 7.76, 6.58, 6.28 ▪ R: 7.76, 6.28
Occlusion velocity (mm/s) ▪ Stimulated = 36 ± 1.7 ▪ Baseline = 1.4 ± 1.1	0	1.55	10.80 (S1: 10.80)	12.98 for all	12.98 for all
Syringe displacement (mm)	0	11.05	18.6771 (S1: 12.45)	▪ A: 20.54, 17.09, 14.67 ▪ R: 20.54, 14.67	▪ A: 20.54, 17.09, 14.67 ▪ R: 20.54, 14.67
Syringe velocity (mm/s)	0	1.97	31.13	38 for all	38 for all
Wave velocity (cm/s) ▪ Stimulated A = 2.2 ± 3.3 ▪ Stimulated R =: 2.2 ±1.8 ▪ Baseline = 0.98 (A only)	0	0.37	3.72 A 3.62 R	▪ A: 4.25, 5.05, 5.26 ▪ R: 4.25, 5.14	▪ A: 4.76, 5.31, 5.54 ▪ R: 6.09, 5.54
Propagation distance (cm) ▪ Stimulated A = 5.3 ± 1.4 ▪ Stimulated R = 6.6 ± 2.2 ▪ Baseline = 3.9 (A only)	0	8.4	28	▪ A: 11.2, 8.40, 8.40 ▪ R:11.20, 11.2	▪ A:5.60, 8.40, 8.40 ▪ R:11.20, 8.40
Cyclic frequency (mins) ▪ (2–6 min [13])	-	2	2	2	2

**Table 3:** Comparison of the existing motility index and the MI_DCM_ from the motility patterns applied to the DCM.

Pattern	MI [Segment × s]	*MI_DCM_*
Static	0	0
Baseline	180	0.0033
CPPW	200	0.016
Stimulated (PEG)	440	0.085
Stimulated (maltose)	380	0.073

**Table 4:** Power law model fitted to release profiles in the DCM under different motility patterns. The constants are presented as mean (95% confidence interval) and relevant regression statistics are presented including root mean square error (RMSE), adjusted correlation coefficient (Adj. R^2^) and standard square error (SSE).

Motility Pattern	*K*	*m*	*R* ^2^	RMSE	SSE
Static	4.51 (3.95, 5.06)	0.77 (0.72, 0.81)	0.999	0.98	9.47
Baseline	5.58 (4.65, 6.51)	0.74 (0.68, 0.80)	0.999	1.567	24.57
Antegrade	5.14 (4.65, 5.63)	0.80 (0.76, 0.83)	0.999	0.90	8.17
Retrograde	4.75 (4.47, 5.03)	0.83 (0.81, 0.85)	0.999	0.55	3.07
Stimulated (PEG)	6.42 (5.76, 7.08)	0.86 (0.82, 0.89)	0.999	1.29	16.74
Stimulated (maltose)	5.85 (5.11, 6.59)	0.90 (0.84, 0.94)	0.999	1.59	25.30

**Table 5:** Release (Q) comparison at t = 4, 10 and 24 h in the DCM using different motility patterns and the USPII at 25, 50 and 100 rpm, presented in the grey boxes. Comparison is colour-coded wherein red indicates > 50% discrepancy between the DCM and the USPII, blue and yellow indicate 10–50% positive and negative difference, respectively and green shows results that lie within 10% of one another. The arrow indicates the positive and negative difference wherein a ‘up’ arrow (↑) shows a positive difference where release in the DCM was higher than the USPII, and vice versa. Statistical similarities where *p* > 0.05 are indicated at each timepoint by values that share the same superscript letter.

	DCM	USPII		
		25 rpm	50 rpm	100 rpm
**Q_4_**				
**USPII**	-	18.97 ^d^	24.20	32.81
**Static**	12.21 ^a^	↓ −35.64%	↓ −49.55%	↓ −62.79%
**Baseline**	15.68 ^b^	↓ −17.34%	↓ −35.21%	↓ −52.21%
**CPPW (Antegrade)**	15.52 ^b^	↓ −18.19%	↓ −35.87%	↓ −52.70%
**CPPW (Retrograde)**	15.69 ^b^	↓ −17.29%	↓ −35.17%	↓ −52.18%
**Stimulated (PEG)**	21.42 ^c^	↑ +12.92%	↓ −11.49%	↓ −34.72%
**Stimulated (maltose)**	20.02 ^c,d^	↑ +5.54%	↓ −17.27%	↓ −38.98%
**Q_10_**				
**USPII**	-	41.11 ^h^	47.95 ^g^	64.10
**Static**	27.29 ^e^	↓ −33.62%	↓ −43.08%	↓ −57.43%
**Baseline**	31.68 ^f^	↓ −22.94%	↓ −33.93%	↓ −50.58%
**CPPW (Antegrade)**	31.65 ^f^	↓ −23.01%	↓ −33.99%	↓ −50.62%
**CPPW (Retrograde)**	32.84 ^f^	↓ −20.12%	↓ −31.51%	↓ −48.77%
**Stimulated (PEG)**	43.40 ^g,h^	↑ +5.57%	↓ −9.49%	↓ −32.29%
**Stimulated (maltose)**	45.26 ^g^	↑ +10.09%	↓ −5.61%	↓ −29.39%
**Q_24_**				
**USPII**	-	73.84 ^k^	101.15 ^m^	95.54 ^m^
**Static**	49.98	↓ −32.31%	↓ −50.59%	↓ −47.69%
**Baseline**	56.74 ^i^	↓ −23.16%	↓ −43.91%	↓–40.61%
**CPPW (Antegrade)**	63.17 ^i,j^	↓ −14.45%	↓ −37.55%	↓ −33.88%
**CPPW (Retrograde)**	68.47 ^j,k^	↓ −7.27%	↓ −32.31%	↓ −28.33%
**Stimulated (PEG)**	91.60 ^m^	↑ +24.05%	↓ −9.44%	↓ −4.12%
**Stimulated (maltose)**	96.74 ^m^	↑ +31.01%	↓ −4.36%	↑ +1.26%

## Data Availability

The data that support the findings of this study and code used for the simulations are freely available on request from the corresponding author.

## Appendix A. Motility Index

Using the original method developed by Marciani et al. [14] (Equation (6)) to quantify the mimicked baseline pattern as an example; there is one single contraction in each of the first 20 s windows, 2 segments contracting as part of an antegrade wave in the third interval and again in the 4th, followed by another isolated contraction in each of the 5th and 6th intervals. This yields a motility index of 180 [segments × s]. In the CPPW, the only activity is from all segments in the first window, yielding an MI of [200 segments × s].

**Baseline**

$$MI=\left(1\times 20\right)+\left(1\times 20\right)+\left(2\times 20\right)+\left(2\times 20\right)+\left(1\times 20\right)+t_{iv}\left(1\times 20\right)$$

MI=180 [segments × s]

**CPPW**

MI=(10×20)+(0×20)+(0×20)+(0×20)+(0×20)+(0×20)

MI=200 [segments × s]

**Stimulated (PEG)**

MI=(5×20)+(4×20)+(5×20)+(1×20)+(3×20)+(4×20)

MI=440 [segments × s]

**Stimulated (Maltose)**

MI=(5×20)+(2×20)+(4×20)+(1×20)+(3×20)+(4×20)

MI=380 [segments × s]

### Appendix A.1. Specific Motility Index (SMI)

Schutt et al. [16] developed several models of the proximal colon that varied in size and number of segments. To enable comparison of MI between these models, the index was normalised by the number of segments in the model, arriving at the specific MI (Equation (A1)). In vivo, this may hold value for comparing motility between populations, such as paediatric versus adult.

(A1) $$SMI=\frac{\sum_{k=1}^{K}{\left(t_{iv}\cdot N_{seg}\right)}_{k}}{N._{seg,\;total}}$$

The present study applied different motility patterns inside the DCM, wherein the geometry was constant. Characterisation of motility using the specific MI was therefore not appropriate.

### Appendix A.2. DCM Motility Index i (MI_DCMi_)

The number of segments, *N*_seg_, used by Marciani et al. [14] was also derived from analysis of MRI data and depended on visible motility and interpatient variability. For example, the ascending colon was considered to be one segment. Using in vitro and in silico models, the number of segments is well-defined in the geometry of the system and does not rely on imaging capabilities and unpredictable in vivo motility to distinguish between segments. Since parameters that drive motion of the luminal contents, such as occlusion rate and duration of a contraction, are predefined in vitro, as opposed to in vivo situation (which is limited by accessibility of the colon and the current limitations of in vivo imaging techniques), a more precise version of the motility index, MI_DCM_, can be introduced to enable quick and easy comparison of motility patterns. This is a necessary development due to downfalls of the existing methodology. Firstly, discrepancies could be introduced to the calculation when a single contractile episode spans more than one time interval. This can be overcome by replacing t_iv_ with the known duration of a contractile episode, t_c_, as in Equation (A2), calculating MI_DCM,i_ (an iteration of the MI_DCM_). In this case, K becomes the total number of motility episodes in the 120 s window.

(A2) $$MI_{DCM,\;i}=\overset{K}{\underset{k=1}{\sum }}{\left(t_{c}\cdot N_{seg}\right)}_{k}$$

However, this highlights the issue of using a temporal expression, since an unfairly heavy weighting is awarded to slow contractions that are less likely to generate higher luminal pressures, flows and intense mixing. For example, the MI_DCM,i_ calculated for the PEG and maltose stimulated motility using this methodology was 81.17 and 26.42 compared to the baseline MI of 80.52 which is clearly misleading:

***Baseline***

$$MI_{DCMi}=\left(2.94\times 1\right)+\left(2.94\times 1\right)+\left(22.92\times 3\right)+\left(2.94\times 1\right)+\left(2.94\times 1\right)$$

$$MI_{DCMi}=80.52\;$$

***CPPWs***

$$MI_{DCMi}=\left(10\times 13.88\right)$$

$$MI_{DCMi}=138.8\;$$

***Stimulated (PEG)***

$$\begin{array}{ll} MI_{DCMi,\;\;\;PEG}= & \left(4\times 2.64\right)+\left(1\times 0.53\right)+\left(4\times 4.25\right)+\left(1\times 0.53\right)+\left(4\times 5.14\right)+\left(1\times 0.53\right)+\left(3\times 5.05\right)\\ & +\left(1\times 0.53\right)+\left(3\times 5.26\right) \end{array}$$

$$MI_{DCM,\;\;\;PEG}=81.17\;$$

***Stimulated (Maltose)***

$$MI_{DCMi,\;\;\;maltose}=\left(4\times 1.84\right)+\left(2\times 1.58\right)+\left(3\times 1.52\right)+\left(3\times 1.58\right)+\left(3\times 1.52\right)+\left(4\times 0.51\right)$$

$$MI_{DCMi,\;\;\;maltose}=26.42\;$$

This highlights the downfall of this calculation; that the duration of the motility increases inversely with occlusion velocity, therefore a falsely high weighting is awarded to slower waves.

### Appendix A.3. DCM Motility Index (MI_DCM_)

Stamatopoulos et al. [6] and Arkwright et al. [19] demonstrated occlusion velocity to be key in generating pressure during non-occluding events (more important than occlusion degree). Therefore, the index was further developed by replacing the duration of a contraction t_c_ with occlusion velocity, v. A critical benefit of the existing Marciani et al. [14] methodology was that the summative nature of the calculation inherently increases MI with the frequency of contractile activity, which Schutt et al. [16] reported to be an effective predictor of dissolution ability. To further increase discriminatory power of the MI_DCM_ between motility patterns that may have different repetition frequencies, MI_DCM_ was normalised by the frequency, 120 s for all patterns in this study. Therefore, the resulting MI (Equation (A3). Equation (7) in main text) is sensitive to occlusion velocity, frequency of occluding events and the number of segments involved in a contractile episode. Finally, to non-dimensionalise the index, the number of segments was divided by the length of a segment, L.

**Baseline**

(A3)
$$MI_{DCM}=\frac{1}{\omega \cdot L}\overset{K}{\underset{k=1}{\sum }}{\left(v\cdot N_{seg}\right)}_{k}$$

$$MI_{DCM}=\frac{1}{120\times 28}\left[\left(1\times 1.6358\right)+\left(1\times 1.6358\right)+\left(3\times 1.55\right)+\left(1\times 1.6358\right)+\left(1\times 1.6358\right)\right]\;$$

$$MI_{DCM}=0.0033$$

**CPPWs**

$$MI_{DCM}=\frac{1}{120\times 28}\left[\left(1\times 5.4\right)+\left(9\times 5.40\right)\right]$$

$$MI_{DCM}=0.016\;$$

**Stimulated (PEG)**

$$\begin{array}{ll} MI_{DCM}= & \frac{1}{120\times 28}[\left(4\times 12.98\right)+\left(1\times 12.98\right)+\left(4\times 12.98\right)+\left(1\times 12.98\right)+\left(4\times 12.98\right)+\left(1\times 12.98\right)\\ & +\left(3\times 12.98\right)+\left(1\times 12.98\right)+\left(3\times 12.98\right)] \end{array}$$

$$MI_{DCM}=0.085\;$$

**Stimulated (Maltose)**

$$\begin{array}{ll} MI_{DCM}= & \frac{1}{120\times 28}[\left(4\times 12.98\right)+\left(1\times 12.98\right)+\left(2\times 12.98\right)+\left(1\times 12.98\right)+\left(3\times 12.98\right)+\left(1\times 12.98\right)\\ & +\left(3\times 12.98\right)+\left(1\times 12.98\right)+\left(3\times 12.98\right)] \end{array}$$

$$MI_{DCM}=0.074$$

The MI_DCM_ discriminated more between the baseline and CPPW patterns which is important given the differences in occlusion rate, which was dampened using the MI from the existing methodology. Since the motility index increased with occlusion velocity, frequency of pressure-generating events and number of segments involved, this index was considered to be sufficient for this study. In this iteration of motility index, equal weighting is given to single, isolated contractions, as to a contraction that is part of a propagating wave. However, it is likely that the additional relaxation step prior to contraction and the progressive contractions lends a heightened hydrodynamics effect and should be weighted more heavily. However, until this data is available, adjustment may be somewhat arbitrary, so this was considered to be beyond the scope of this work. This provides an insight into a possible representation of in vivo MI in the future as imaging capabilities develop.
